# Genome sequencing and *de novo* assembly of *Trichoderma longibrachiatum* isolate collected from Florida agricultural soils

**DOI:** 10.1128/mra.00906-23

**Published:** 2023-12-11

**Authors:** Andrea Karina Suazo Tejada, Pei-Ling Yu, Katherine E. Smith, Jose C. Huguet-Tapia, Joseph Carrillo, Jeremy T. Brawner, Gary E. Vallad

**Affiliations:** 1 Department of Plant Pathology, Gulf Coast Research and Education Center, University of Florida, Wimauma, Florida, USA; 2 Department of Plant Pathology, University of Florida, Gainesville, Florida, USA; 3 United States Department of Agriculture Forest Service, Southern Institute of Forest Genetics, Saucier, Mississippi, USA; University of California, Riverside, Riverside, California, USA

**Keywords:** *Trichoderma longibrachiatum*, genome, biocontrol

## Abstract

We report a draft genome assembly of *Trichoderma longibrachiatum* isolate GEV 3550, obtained from Florida, United States of America.

## ANNOUNCEMENT

*Trichoderma* belongs to the phylum Ascomycota and the family Hypocreaceae; it is found in different types of soils and rotting wood (1). Some *Trichoderma* spp. are beneficial and can be used as biological control agents (2). Isolate GEV 3550 was isolated from a compost pile destined for application to a vegetable production field in Manatee County, Florida, USA. One gram of the sample was diluted and plated on modified potato dextrose agar (PDA) (BD Difco, Detroit, MI, USA) (3) at 28°C for 3 days. Individual colonies were transferred to PDA and grown for 7 days. The isolate was identified as *T. longibrachiatum* (4), based on the sequence of the DNA-directed RNA polymerase II subunit and translation elongation factor 1 (5). The isolate was assessed at varying physical parameters and was identified to be tolerant to high temperatures, making it an ideal biological control agent for use in warmer climates.

To perform the whole-genome sequence, the isolate was cultured on PDA overlaid with cellophane (Research Products International, IL, USA) for 5 days (6), and fungal hyphae were harvested and lyophilized overnight. High molecular weight (HMW) DNA was extracted using a modified edition of a previously described protocol (7). The overall integrity of HMW DNA was analyzed by TapeStation (Agilent 4200). Size peaked at 57.978 kb, and DNA integrity number = 8.9 indicates that the HMW DNA is of high quality. To eliminate DNA fragments shorter than 10 kb, a short-read eliminator kit SRE XS (SKU 102-208-200) was used (Circulomics, Inc., Baltimore, MD, USA). One microgram of HMW DNA was used to prepare the sequencing library using the ligation sequencing kit (SQK-LSK109; Oxford Nanopore Technologies, Oxford, UK) following the manufacturer’s instructions. After sequencing adapter ligation, the library was enriched for DNA fragments that are longer than 3 kb using long-fragment buffer. The library was sequenced on an R9.4.1 flow cell using a MinION device (Oxford Nanopore Technologies) for 72 hours. Base calling was completed with Guppy 4.4.1 (8). The quality of the reads was evaluated using NanoPlot 1.30.1 (9), and a total of 411,350 raw reads were generated with a read length N50 of 14,499 bp from the Nanopore sequencing. Moreover, Porechop 0.2.4 was used to remove adaptors added in the library preparation (10). Additionally, reads shorter than 1,000 bp and quality scores lower than 8 were removed using Filtlong 0.2.0 (11). The quality of the filtered reads was assessed with NanoPlot, and a total of 357,782.0 reads were retained. Additionally, the isolate’s DNA sample was sent to SeqCenter (Pittsburgh, PA, USA) for Illumina sequencing. The Illumina DNA Prep Kit and integrated DNA technologies (IDT) 10 bp unique dual indexes (UDI) were used for library preparation and sequenced on an Illumina NextSeq 2000, producing 2 × 151 bp reads, resulting in a total of 14,955,415 paired-end reads. Demultiplexing, quality control, and adaptor trimming were performed with BCL Convert 3.9.3 (12). The sequencing depth of Nanopore and Illumina sequencing was 98× and 129×, respectively.

*De novo* sequence assembler was deployed to assemble filtered Nanopore reads, Flye 2.9.1 (13). The draft assembly was first polished by Medaka 1.6.0 (https://github.com/nanoporetech/medaka) using filtered Nanopore reads and then further polished twice by mapping Illumina short reads to the polished assemblies using Pilon 0.7.17 (14). Finally, we used Inspector 2.1.1 (15) to evaluate the polished assembly. The quality of the assembly generated was evaluated via QUAST 5.0.2 (16). The final assembly has a total size of 31.5 Mb. The Nanopore sequencing coverage was 89×. The assembly completeness was assessed with Benchmarking Universal Single-Copy Orthologs (BUSCO) 5.4.4 (17) with the Hypocreales_odb10 lineage data set, and completeness was 99.3% with a total of 4,494 BUSCO markers (Table 1).

**TABLE 1:** Comparison of *Trichoderma longibrachiatum* GEV3550 and reference genome strain-L4

Characteristic	*T. longibrachiatum* GEV3550	*T. longibrachiatum* strain-L4
Isolation source	Compost	Soil
Sequencing technology	Oxford Nanopore + Illumina	PacBio
No. of contigs	15	11
Genome assembly size (Mb)	31.5	32.4
%GC	53.8%	53%
Largest contig (bp)	4,247,245	6,479,932
N 50 (bp)	3,213,272	4,957,914
L50 (bp)	5	3
Complete BUSCOs (%)	99.3%	99.3%
Complete BUSCOs (C)	4,463	4,461
Complete and single-copy BUSCOs (S)	4,459	4,456
Complete and duplicated BUSCOs (D)	4	5
Fragmented BUSCOs (F)	1	2
Missing BUSCOs (M)	30	31
Total BUSCOs	4,494	4,494
Bioproject accession no.	PRJNA975852	PRJNA885614

This study will aid the future study of the biology behind the high-temperature tolerance and promising biocontrol activity of *T. longibrachiatum*.

## Data Availability

The described whole-genome assembly is deposited under BioProject accession number PRJNA975852. The Illumina and Oxford Nanopore reads are deposited at the Sequence Read Archive (SRA) under accession numbers SRX20592107 and SRX20592106, respectively.
